# Incidence and pattern of childhood cancer in Addis Ababa, Ethiopia (2012–2017)

**DOI:** 10.1186/s12885-023-11765-7

**Published:** 2023-12-21

**Authors:** Amanuel Belay, Ahmed Ali, Wondimu Ayele, Mathewos Assefa, Ahmedin Jemal, Eva J. Kantelhardt

**Affiliations:** 1 Centre for Innovative Drug Development and Therapeutic Trials for Africa (CDT- Africa), Addis Ababa University, Addis Ababa, Ethiopia; 2 School of Public Health, College of Health Sciences, Addis Ababa University, Addis Ababa, Ethiopia; 3 Department of Oncology, School of Medicine, Addis Ababa University, Addis Ababa, Ethiopia; 4 American Cancer Society, Atlanta, GA USA; 5 Department of Gynecology, Martin Luther University Halle-Wittenberg, Halle (Saale), Germany; 6 Institute of Medical Epidemiology, Biostatistics and Informatics, Martin Luther University Halle-Wittenberg, Halle (Saale), Germany

**Keywords:** Childhood cancer, Incidence, Pattern, Addis Ababa, Ethiopia

## Abstract

**Background:**

Cancer is becoming a major public health problem globally and a leading cause of death in children in developed countries. However, little is known about the epidemiology of childhood cancer in Ethiopia. This study, therefore, assessed childhood cancer incidence patterns in Addis Ababa using the Addis Ababa city population-based cancer registry data from 2012 to 2017.

**Methods:**

Invasive cancer cases diagnosed in ages 0–14 years from 2012 to 2017 were obtained from the Addis Ababa City population-based Cancer Registry. Cases were grouped according to the International Classification of Childhood Cancer, 3rd edition (ICCC-3) based on morphology and primary anatomic site. Age-standardized incidence rates (ASR) were calculated by the direct method using the world standard population.

**Results:**

The overall average annual incidence rate during 2012–2017 in children was 84.6 cases per million, with rates higher in boys (98.97 per million) than in girls (69.7 per million). By age, incidence rates per million increased from 70.8 cases in ages 0–4 years to 88.4 cases in ages 5–9 years to 110.0 cases 10–14 years. Leukaemia was the most common childhood cancer in both boys (29.1%) and girls (26.8%), followed by lymphoma in boys (24.7%) and renal tumours (13.1%) in girls. The overall cancer incidence rate decreased from 87.02 per million in 2012 to 51.07 per million in 2017.

**Conclusion:**

The burden of childhood cancer is considerably high in Addis Ababa. The observed distribution of childhood cancer in Addis Ababa differs from other African countries. This study highlights the need for further research and understanding of the variations in cancer patterns and risk factors across the region.

**Supplementary Information:**

The online version contains supplementary material available at 10.1186/s12885-023-11765-7.

## Introduction

Childhood and adolescent cancers contribute significantly to the global cancer burden [1]. Childhood cancer incidence has increased by 13% globally [2]. Each year more than 300,000 Children and adolescents are diagnosed with cancer worldwide. [3]. Of children who develop cancer, approximately 90% occur in low- and middle-income countries More than 80% of diagnosed cases of childhood cancer occur in low-income and middle-income countries [4]. Due to limited access to diagnostics and treatment [5] the survival rates are less than 30% in lower-middle-income and low-income countries compared to over 80% in high-income countries (HIC) [6].

The Addis Ababa cancer registry, established in 2011, is the only population-based cancer registry in the country, and childhood cancer cases account for about 4.6% of the total cases [7]. According to clinical record findings from Tikur Anbessa Specialized Hospital, which hosts the largest oncologyreferral and radiotherapy facility in the country [8], most childhood cancer cases present at a late stage of the disease when the chance of survival is poor [9].

Few studies reported on childhood cancer patterns in Ethiopia. Further, all these studies were based on a medical chart review from a single institute rather than a population-based cancer registry [10–12]. Ethiopia has adopted a comprehensive National Action Plan for the Prevention and Control of Chronic Non-Communicable Diseases, and the expansion of cancer treatment services is underway [9]. And epidemiological data are important for estimating and proper allocation of resource needs and health policy prioritization [1].

Therefore, this research has used population-based cancer registry data from Addis Ababa to assess the incidence rate and pattern of childhood cancer in Addis Ababa city. Findings from this research can be used to plan cancer control actions, health care, and allocation of resources for developing effective programs aiming at the control, prevention, diagnosis, and treatment guidelines of childhood cancer in Ethiopia.

## Methods

### Study setting

The study was conducted in Addis Ababa city. Addis Ababa is the capital and largest city of Ethiopia. Addis Ababa has a population of 3,435,028 people (as of the 2017 population projection) [13]. It is a rapidly growing city, with a young population. About 26% of the population in Addis Ababa is made up of individuals under the age of 15 [13]. Residents of the sub-cities are ethnically and socioeconomically diverse.

### Study design and data sources

In this population-based registry study, Invasive cancer cases diagnosed in ages 0–14 years from 2012 to 2017 were obtained from the Addis Ababa City Cancer Registry. Invasive cancer is a type of cancer that has spread from its original site to other parts of the body. It occurs when cancer cells break through the walls of the tissue where they started and invade nearby healthy tissue. Invasive cancer can also spread to other areas of the body through the bloodstream or lymphatic system [14]. The Addis Ababa City Cancer Registry is the first population-based Cancer Registry (AAPBCR) in the country and it was established in September 2011 at Radiotherapy Center, Tikur Anbessa Specialized Hospital, School of Medicine and Addis Ababa University. The registry actively collects all new cancer patients who are residents of Addis Ababa from 20 collaborating institutions (pathology, oncology and radiotherapy facilities). The AACR unit consists of three full-time employees and has trained 20 contact persons in each health institution that provides cancer diagnostic and treatment services. These contact persons follow a standardized format developed by the International Agency for Research on Cancer and collect data on cancer patients of all ages daily from inpatient and outpatient departments, including pathology units. Cases are reported weekly to the AACR unit and supervised for quality [15, 16]. The population at risk was obtained from the Ethiopian central statistical agency.

### Statistical analysis

The diagnosis was classified according to the 3rd edition of the International Classification of Diseases for Oncology (ICCC-3) [17]. Age-specific incidence rates (ASR) were calculated for three 5-year age groups (0–4 years, 5–9 years, and 10–14 years). Incidence rates were calculated as the average annual number of cases per million child years. Age-standardized rates (ASR) were calculated by the direct method using the world standard population [18]. Incidence of sex ratios was calculated by dividing the incidence among males by that in a female.

Statistical analyses were done using Stata/SE (version 14) [19].

## Results

During the period 2012–2017, 395 childhood cancers were diagnosed in Addis Ababa, Ethiopia. Two hundred twenty-seven (57.5%) of the patients were males and 168 (42.5%) of the patients were females. The median age was 6 (IQR, 3–11). A majority (39.2%) of the patients were between the age group of 0–4 years, followed by 10–14 (31.1%) years age group and 5–9 (29.6%) years age group respectively.

Leukaemias were the most common type of cancer diagnosed among Addis Ababa children, with an incidence of 111 (28.1%). The second most common childhood cancers were Lymphomas, with a frequency of 75(19%), followed by Soft tissue and other extraosseous sarcomas with 51(12.9%), Renal Tumors with 39 (9.9%) and other malignant epithelial neoplasms and malignant melanomas with 28 (7.1%).

Childhood cancer types were found different for males and females (Table 1; STable 1). In males, Leukemias (29.1%) were the most common cancer followed by Lymphomas (24.7%), Soft tissue and other extraosseous sarcomas (13.7%), Malignant bone tumours (7.5%) and Other malignant epithelial neoplasms and malignant melanomas (6.2%) respectively. In contrast, in females, leukaemias (26.8%) were the most common cancer followed by Renal tumours (13.10%), Soft tissue and other extraosseous sarcomas (11.9%), Lymphomas (11.3%) and Other malignant epithelial neoplasms and malignant melanomas (8.3%).

**Table 1:** Number of childhood cancer in Addis Ababa from 2012–2017 by diagnostic group of the International Classification of Childhood Cancer-third edition and sex

Types of malignancy	Male	Female	Total
	N(%)	N(%)	N(%)
All cases	227(100.0)	168(100.0)	395(100.0)
Leukemias, myeloproliferative diseases, and myelodysplastic diseases	66(29.0)	45(26.8)	111(28.1)
Lymphomas and reticuloendothelial neoplasms	56(24.7)	19(11.3)	75(19)
Soft tissue and other extraosseous sarcomas	31(13.7)	20(11.9)	51(12.9)
Renal tumours	12(5.3)	22(13.1)	39(9.9)
Other malignant epithelial neoplasms and malignant melanomas	14(6.2)	14(8.3)	28(7.1)
Malignant bone tumours	17(7.5)	9(5.4)	26(6.6)
Retinoblastoma	7(3.1)	12(7.1)	19(4.8)
CNS and miscellaneous intracranial and intraspinal neoplasms	7(3.1)	6(3.6)	13(3.3)
Germ cell tumours, trophoblastic tumours, and neoplasms of gonads	1(0.4)	11(6.5)	12(3.0)
Neuroblastoma and other peripheral nervous cell tumours	3(1.3)	7(4.2)	10(2.5)
Other and unspecified malignant neoplasms	5(2.2)	2(1.2)	7(1.8)
Hepatic tumours	3(1.3)	1(0.6)	4(1.0)

The range of tumour types varied markedly with age groups (Table 2). In the age group 0–4, Leukemias were the most common cancer representing 26% of all cases. However the proportion was higher in the age group 5–9 representing 32.5% of cases but, in the age group 10–14, the proportion of Leukemias was 22.8% of all cases. Renal tumours were the second most frequent tumours in the age group 0–4 representing 16.8% of cases however the proportion of Renal tumour cases was 7.7% and 3.3% in the age group 5–9 and 10–14 respectively. Soft tissue sarcomas were the third most common cancer in the age group 0–4 (13.5%) and 5–9 (11.1%). Bone tumours represented 0.6% of all cases in the age group but were the third most common cancer in the age group 10–14.

**Table 2:** Number of childhood cancer in Addis Ababa from 2012–2017 by diagnostic group of the International Classification of Childhood Cancer-third edition and age group

Diagnosis group	Age group		
	0–4	05–09	10–14
	N (%)	N (%)	N (%)
All cases	155 (39.2)	117 (29.6)	123 (31.1)
Leukemias, myeloproliferative diseases, and myelodysplastic diseases	45 (11.4)	38 (9.6)	28 (7.1)
Renal tumours	26 (6.6)	9 (2.3)	4 (1.0)
Soft tissue and other extraosseous sarcomas	21 (5.3)	13 (3.3)	17 (4.3)
Lymphomas and reticuloendothelial neoplasms	18 (4.6)	29 (7.3)	28 (7.1)
Retinoblastoma	18 (4.6)	1 (0.3)	0
Other malignant epithelial neoplasms and malignant melanomas	10 (2.5)	4 (1.0)	14 (3.5)
Neuroblastoma and other peripheral nervous cell tumours	4 (1.0)	5 (1.3)	1 (0.3)
Germ cell tumours, trophoblastic tumours, and neoplasms of gonads	4 (1.0)	1 (0.3)	7 (1.8)
Other and unspecified malignant neoplasms	4 (1.0)	2 (0.5)	1 (0.3)
CNS and miscellaneous intracranial and intraspinal neoplasms	3 (0.8)	5 (1.3)	5 (1.3)
Hepatic tumours	1 (0.3)	3 (0.8)	0
Malignant bone tumours	1 (0.3)	7 (1.8)	18 (4.6)

The overall incidence rate of childhood cancer from 2012 to 2017 was 84.6 cases per million with an average of 65.8 cases per year. The incidence rate in boys was 98.97 cases per million and that of girls was 69.7 cases per million. The sex ratio for all childhood cancer incidence was 1.42. The incidence rate was higher among children aged 10–14 with 100 cases per million followed by children aged 5–9 and 0–4 with 79 cases per million and 76.6 cases per million respectively.

The overall cancer incidence rate decreased from 85.4 per million in 2012 to 50.4 per million in 2017 (Table 3). The incidence rates were higher among boys than girls from 2012 to 2017. Age group 10–14 had the highest incidence rate in both 2012 and 2017. However, the incidence rate was higher among the age group 5–9 in the year 2013 (124.3 per million). Leukemia’s had the highest incidence rate in both the years 2012 and 2017 (Table 4). Meanwhile, in the year in the year 2015, Lymphoma’s had the highest incidence rate with 17.5 per million.

**Table 3:** Childhood cancer incidence by sex and age group from 2012–2017

	2012		2013		2014		2015		2016		2017	
	N	ASR	N	ASR	N	ASR	N	ASR	N	ASR	N	ASR
Over all	62	85.4	80	106.4	86	108.8	63	76.8	60	71.7	44	50.4
Male	36	102.8	49	132.7	45	116.2	35	86	30	72.8	32	73.6
Female	26	68.3	31	80.3	41	102.4	28	67.7	30	70.8	12	27.5
Age group												
00–04	22	68.8	31	93.9	37	108.5	27	76.6	20	54.9	18	47.8
05–09	17	85.9	27	124.3	23	96.4	18	68.8	20	69.6	12	37.6
10–14	23	106.9	22	103.2	26	123.0	18	85.9	20	96.3	14	68.0

^a^ASR = Age Standardized Incidence Rate

**Table 4:** Childhood cancers incidence by diagnostic group of the International Classification of Childhood Cancer-third edition

Specific cancer type	2012		2013		2014		2015		2016		2017	
	**N**	**WSR**	**N**	**WSR**	**N**	**WSR**	**N**	**WSR**	**N**	**WSR**	**N**	**WSR**
Leukemias	16	23.3	21	27.8	29	36.1	13	16.1	20	23.5	12	12.2
Lymphoma	12	17.6	21	28.3	12	16.1	14	17.5	10	13.0	6	6.5
Soft tissue sarcomas	10	13.2	10	13.5	9	12.3	7	8.1	6	6.5	9	11.2
Renal tumours	8	9.8	8	9.9	6	6.8	9	11.0	6	6.6	2	2.0
Epithelial tumours and melanoma	5	6.3	2	2.5	3	3.6	7	8.8	6	7.9	5	6.8
Other^b^	11	15.1	18	24.4	27	33.9	13	15.5	12	14.3	10	11.8

^a^ASR = Age Standardized Incidence Rate

^b^Other = Bone tumours, Retinoblastoma, CNS tumours, Germ cell and gonadal tumours, Sympathetic nervous system, Other and unspecified, and Hepatic tumours

## Discussion

This is the first population-based childhood cancer study done in Ethiopia. The key findings from this study showed that the overall incidence rate of childhood cancer from 2012 to 2017 was 81.9 cases per million.

In Addis Ababa, the most common cancer was leukaemias followed by Lymphoma and Soft tissue and other extraosseous sarcomas respectively. In contrast to our finding at Tikur Anbessa Specialized Hospital, the most common malignancies were Wilm’s tumour (24.7%) followed by leukaemia (19.5%) and lymphomas (14.3%) respectively from January 2005 to December 2006 [12]. And at Gondar University Hospital, 60.6% of the reported childhood cancers were haematological malignancies, followed by Wilms tumour [11]. Another study in Gonder Hospital showed that the most common malignancies in children to be Lymphoma, Wilma’s tumour, and Retinoblastoma respectively [10]. The observed difference in the incidence of leukaemia might be due to, the difficulty in diagnosing. Leukaemia can cause various symptoms that can be mistaken for other conditions, such as infection. This can lead to early death if the cancer is not suspected or diagnosed in time [20].

The observed distribution was different compared to some African countries. The most common childhood cancer in Africa are Lymphomas, nephroblastoma, Kaposi sarcoma, and retinoblastoma [21]. These cancers are often related to infections, such as malaria, Epstein-Barr virus (EBV), HIV/AIDS, and human herpesvirus 8 (HHV8) [22, 23]. Similar to our findings, Leukaemias are reported as the most common childhood cancer in Tunisia [24] and Namibia [25]. In contrast to our finding Burkitt lymphoma is the most commonly diagnosed childhood cancer in Malawi [26]. Lymphomas were the second most common cancer in Addis Ababa, however, Wilms’ tumour and retinoblastomas are the second most common cancers in Malawi [26] and Namibia [25] respectively.

Globally, Brain tumours are the second most common childhood cancer [2]. Similarly, findings from other countries show brain tumours to be the second most common [27–29] or the third most common malignancy [24, 30–32] in children. In contrast, CNS cancer is the eighth most common cancer in Addis Ababa. In Ethiopia, brain tumours represent only 3% of diagnosed childhood cancers [16]. Brain tumours are difficult to diagnose in Africa because of a lack of access to specialized diagnostic tools and the high cost of treatment [21, 22]. In addition, brain tumours can be misdiagnosed as other conditions. This is because the symptoms of brain tumours can be similar to the symptoms of other conditions, such as cerebral malaria, tuberculous meningitis, or bacterial meningitis [21].

In Addis Ababa, the overall age-standardized incidence rates of childhood cancer were 81.9 cases per million. The observed childhood cancer incidence in this study was higher than the incidence observed in South Africa (45.7 per million) [33] and Namibia (29.4 per million) [25]. However, it was lower when compared to the global childhood cancer incidence rate (140.6 per million) [2] and other countries like Zimbabwe (130.0 per million) [34], Uganda (147.2 per million) [34], Spain (155.8 per million) [28], Thailand (74.9 per million) [31], and Korea (134.9 per million) [35]. The reasons for the difference in the incidence rate are not entirely clear, however, it may be attributable to diversity registration practices, and actual racial and geographical differences [20]. In addition, in low-income countries, where access to healthcare is limited, some children with cancer may not be diagnosed or registered in time to receive treatment [20].

Although the number of children diagnosed with cancer in Africa is not expected to increase, the number of reported cases may rise due to better cancer registries, increased awareness, knowledge, and access to diagnostic tools and treatments. [21]. The trend of childhood cancer is expected to change in Africa due to a decrease in Kaposi sarcoma, a cancer linked with HIV, and an increase in leukaemia, a cancer associated with a higher socioeconomic condition [21, 36]. In this study, a decreasing trend in the incidence of childhood cancer was observed. In contrast to our finding a study done at Tikur Anbessa Spatialized Hospital and Gondar University Hospital showed an increasing pattern in the incidence of childhood cancer [11, 12]. The reasons for decreasing trends in this study are unknown however, the observed changes might be due to, patients being diagnosed at a health facility that is not the source of the cancer registry, misdiagnosis, and lack of quality and affordable diagnostic tools.

Although our study provides valuable insights, it is important to acknowledge several limitations. The registry may not capture all cases of childhood cancer in Addis due to various reasons. First, the registry collects cancer cases from selected institutions in the city, so not all facilities treating patients in the city reports cases to the registry. Second, due to limited access to healthcare, inadequate awareness of cancer, and cultural barriers to seeking care, It’s possible that a significant portion of children with cancer have never received any medical care. As a result, the registry’s data may underestimate the true incidence of cancer in the city. Furthermore, the registry does not follow-up with patients after they are diagnosed with cancer. This makes it difficult to track the progress of patients over time and assess the survival rate of childhood cancer in Ethiopia.

## Conclusion

The overall childhood cancer incidence rate from 2012 to 2017 was 81.9 cases per million. The incidence rate showed a decreasing pattern in Addis Ababa, both in males and females. Although we do not fully understand the reasons for these changes, the pattern seen in this study may be due to random variation or changes in reporting. This research highlights the need for more research into the epidemiology of childhood cancer to improve the quality of care, early detection, and improve the survivorship of childhood cancer. Implementing a more effective and user-friendly electronic data collecting system will assist to enhance the quality of the data for the Addis Ababa cancer registry, which is crucial. The registry should also build effective mechanisms to gather and update patients’ follow-up information on treatment, survival, and recurrence since this information can offer important insights into the course of cancer and the effectiveness of therapy. Moreover, including more facilities to collect cancer cases would expand the registry and offer a more comprehensive view of the pediatric cancer burden in Addis Ababa.

### Electronic supplementary material

Below is the link to the electronic supplementary material.

Supplementary Material 1

## Data Availability

The data that support the findings of this study are available from Addis Ababa Cancer Registry but restrictions apply to the availability of these data, which were used under license for the current study, and so are not publicly available. Data are however available from the corresponding author upon reasonable request and with permission of the Addis Ababa Cancer Registry.

## List of abbreviations

AAPBCR: Addis Ababa City population-based Cancer Registry
ASR: Age-standardized rates
CNS: Central Nerves System
EBV: Epstein-Barr virus
HHV8: Human herpes virus 8
HIV/AIDS: Human Immunodeficiency Virus/Acquired Immune Deficiency Syndrome
ICCC-3: International Classification of Childhood Cancer, 3rd edition
IQR: Interquartile rage
SD: Standard deviation
