# Supplementary material for: Incidence and pattern of childhood cancer in Addis Ababa, Ethiopia (2012–2017)
Source: BMC Cancer. 2023 Dec 21;23:1261. doi: 10.1186/s12885-023-11765-7 (PMC10734044; doi:10.1186/s12885-023-11765-7)
Supplement: Supplementary file 1 — Supplementary Material 1 [file 12885_2023_11765_MOESM1_ESM.pdf]

Supporting information

STable 1: Number of childhood cancer in Addis Ababa from 2012-2017 by diagnostic group of the International Classification of Childhood Cancer-third edition and sex

Diagnostic Group		Female	Male	Total
**Overall**		**168**	**227**	**395**
**I.**	**Leukemias, myeloproliferative diseases, and myelodysplastic diseases**	**45**	**66**	**111**
**a.**	Lymphoid leukaemias	34	52	86
**b.**	Acute myeloid leukaemias	8	5	13
**c.**	Chronic myeloproliferative diseases	3	5	8
**d.**	Myelodysplastic syndrome and other myeloproliferative diseases		2	2
**e.**	Unspecified and other specified leukaemias		2	2
**II.**	**Lymphomas and reticuloendothelial neoplasms**	**19**	**56**	**75**
**a.**	Hodgkin lymphomas	6	16	22
b.	Non-Hodgkin lymphomas (except Burkitt lymphoma)	12	27	39
**c.**	Burkitt lymphoma	1	4	5
**d.**	Miscellaneous lymphoreticular neoplasms		1	1
**e.**	Unspecified lymphomas		8	8
**III.**	**CNS and miscellaneous intracranial and intraspinal neoplasms**	**6**	**7**	**13**
**a.**	Ependymomas and choroid plexus tumours	1		1
b.	Astrocytomas	1	4	5
c.	Intracranial and intraspinal embryonal tumours	2	2	4
d.	Other gliomas		1	1
e.	Other specified intracranial and intraspinal neoplasms	1		1
f.	Unspecified intracranial and intraspinal neoplasms	1		1
**IV.**	**Neuroblastoma and other peripheral nervous cell tumours**	**7**	**3**	**10**
a.	Neuroblastoma and ganglioneuroblastoma	6	2	8
b.	Other peripheral nervous cell tumours	1	1	2
**V.**	**Retinoblastoma**	**12**	**7**	**19**
**VI.**	**Renal tumours**	**22**	**17**	**39**
a.	Nephroblastoma and other nonepithelial renal tumours	21	17	38
b.	Renal carcinomas	1		1
c.	Unspecified malignant renal tumours			
**VII.**	**Hepatic tumours**	**1**	**3**	**4**
**a.**	Hepatoblastoma	1	1	2
b.	Hepatic carcinomas		2	2
c.	Unspecified malignant hepatic tumours			
**VIII.**	**Malignant bone tumors**	**9**	**17**	**26**
**a.**	Osteosarcomas	6	7	13
b.	Chondrosarcomas	1		1
c.	Ewing tumour and related sarcomas of bone		9	9
d.	Other specified malignant bone tumours			
e.	Unspecified malignant bone tumours	2	1	3

**IX.**	**Soft tissue and other extraosseous sarcomas**	**20**	**31**	**51**
**a.**	Rhabdomyosarcomas	8	13	21
b.	Fibrosarcomas, peripheral nerve sheath tumours, and other fibrous neoplasms		3	3
c.	Kaposi sarcoma		1	1
d.	Other specified soft tissue sarcomas	3	7	10
e.	Unspecified soft tissue sarcomas	9	7	16
**X.**	**Germ cell tumours, trophoblastic tumours, and neoplasms of gonads**	**11**	**1**	**12**
a.	Intracranial and intraspinal germ cell tumors			
**b.**	Malignant extracranial and extragonadal germ cell tumours	4	1	5
c.	Malignant gonadal germ cell tumours	3		3
d.	Gonadal carcinomas	3		3
e.	Other and unspecified malignant gonadal tumours	1		1
**XI.**	**Other malignant epithelial neoplasms and malignant melanomas**	**14**	**14**	**28**
a.	Adrenocortical carcinomas	1		1
b.	Thyroid carcinomas		2	2
c.	Nasopharyngeal carcinomas	1		1
d.	Malignant melanomas	1		1
e.	Skin carcinomas	1	3	4
f.	Other and unspecified carcinomas	10	9	19
**XII.**	**Other and unspecified malignant neoplasms**	**2**	**5**	**7**
a.	Other specified malignant tumours	1	1	2
b.	Other unspecified malignant tumours	1	4	5

STable 2: Number of childhood cancer in Addis Ababa from 2012-2017 by diagnostic group of the International Classification of Childhood Cancer-third edition and age group

Diagnostic Group		0-4	10-14	5-9
**Over All**		**155**	**123**	**117**
**I.**	**Leukemias, myeloproliferative diseases, and myelodysplastic diseases**	**45**	**28**	**38**
a.	Lymphoid leukaemias	37	16	33
b.	Acute myeloid leukaemias	2	7	4
c.	Chronic myeloproliferative diseases	4	3	1
d.	Myelodysplastic syndrome and other myeloproliferative diseases		2	
e.	Unspecified and other specified leukaemias	2		
**II.**	**Lymphomas and reticuloendothelial neoplasms**	**18**	**28**	**29**
a.	Hodgkin lymphomas	1	11	10
b.	Non-Hodgkin lymphomas (except Burkitt lymphoma)	13	15	11
c.	Burkitt lymphoma	2	1	2
d.	Miscellaneous lymphoreticular neoplasms		1	
e.	Unspecified lymphomas	2		6
**III.**	**CNS and miscellaneous intracranial and intraspinal neoplasms**	**3**	**5**	**5**
a.	Ependymomas and choroid plexus tumours			1
b.	Astrocytomas	2	2	1
c.	Intracranial and intraspinal embryonal tumours	1	1	2
d.	Other gliomas		1	
e.	Other specified intracranial and intraspinal neoplasms		1	
f.	Unspecified intracranial and intraspinal neoplasms			1
**IV.**	**Neuroblastoma and other peripheral nervous cell tumours**	**4**	**1**	**5**
a.	Neuroblastoma and ganglioneuroblastoma	4	1	3
b.	Other peripheral nervous cell tumours			2
**V.**	**Retinoblastoma**	**18**		**1**
**VI.**	**Renal tumours**	**26**	**4**	**9**
a.	Nephroblastoma and other nonepithelial renal tumours	26	4	8
b.	Renal carcinomas			1
c.	Unspecified malignant renal tumours			
**VII.**	**Hepatic tumours**	**1**		**3**
a.	Hepatoblastoma	1		1
b.	Hepatic carcinomas			2
c.	Unspecified malignant hepatic tumours			
**VIII.**	**Malignant bone tumors**	**1**	**18**	**7**
a.	Osteosarcomas		9	4
b.	Chondrosarcomas		1	
c.	Ewing tumour and related sarcomas of bone	1	5	3
d.	Other specified malignant bone tumours			
e.	Unspecified malignant bone tumours		3	
**IX.**	**Soft tissue and other extraosseous sarcomas**	**21**	**17**	**13**
a.	Rhabdomyosarcomas	12	5	4

b.	Fibrosarcomas, peripheral nerve sheath tumours, and other fibrous neoplasms	1	1	1
c.	Kaposi sarcoma		1	
d.	Other specified soft tissue sarcomas	3	4	3
e.	Unspecified soft tissue sarcomas	5	6	5
**X.**	**Germ cell tumours, trophoblastic tumours, and neoplasms of gonads**	**4**	**7**	**1**
a.	Intracranial and intraspinal germ cell tumors			
b.	Malignant extracranial and extragonadal germ cell tumours	3	1	1
c.	Malignant gonadal germ cell tumours		3	
d.	Gonadal carcinomas	1	2	
e.	Other and unspecified malignant gonadal tumours		1	
**XI.**	**Other malignant epithelial neoplasms and malignant melanomas**	**10**	**14**	**4**
a.	Adrenocortical carcinomas	1		
b.	Thyroid carcinomas		2	
c.	Nasopharyngeal carcinomas		1	
d.	Malignant melanomas	1		
e.	Skin carcinomas	2	2	
f.	Other and unspecified carcinomas	6	9	4
**XII.**	**Other and unspecified malignant neoplasms**	**4**	**1**	**2**
a.	Other specified malignant tumours	1		1
b.	Other unspecified malignant tumours	3	1	1
